# Fundamental Trade-Offs in the Robustness of Biological Systems with Feedback Regulation

**DOI:** 10.1021/acssynbio.4c00704

**Published:** 2025-04-08

**Authors:** Nguyen Hoai Nam Tran, An Nguyen, Tasfia Wasima Rahman, Ania-Ariadna Baetica

**Affiliations:** † Department of Mechanical Engineering and Mechanics, Drexel University, Philadelphia, Pennsylvania 19104, United States; ‡ School of Biomedical Engineering, Science, and Health Systems, Drexel University, Philadelphia, Pennsylvania 19104, United States

**Keywords:** synthetic biology, systems biology, biological feedback, sensitivity analysis, multiobjective optimization

## Abstract

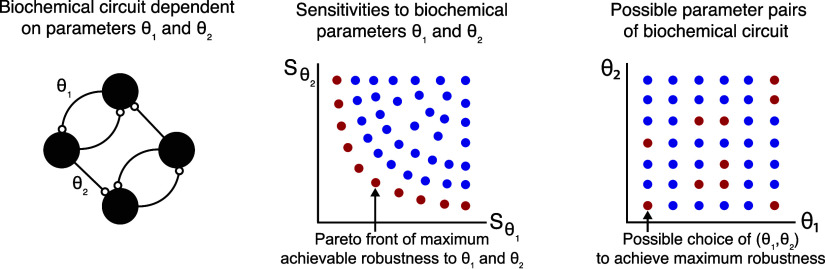

Natural biological systems use feedback regulation to effectively respond and adapt to their changing environment. Even though in engineered systems we understand how accurate feedback can be depending on the electronic or mechanical parts that it is implemented with, we largely lack a similar theoretical framework to study feedback regulation in biological systems. Specifically, it is not fully understood or quantified how accurate or robust the implementation of biological feedback actually is. In this paper, we study the sensitivity of biological feedback to variations in biochemical parameters using five example circuits: positive autoregulation, negative autoregulation, double-positive feedback, positive–negative feedback, and double-negative feedback (the toggle switch). We find that some of these examples of biological feedback are subjected to fundamental performance trade-offs, and we propose multi-objective optimization as a framework to study their properties. The impact of this work is to improve robust circuit design for synthetic biology and to improve our understanding of feedback for systems biology.

## 1 Introduction

In biological systems, feedback can regulate and maintain the stability of the internal environments of organisms and cells, facilitating homeostasis despite changes in their external environments.^1−3^ Additionally, feedback in biological systems can generate dynamic responses such as oscillations, signal amplification, and facilitate rapid switching between multiple states.^4−8^ These roles of biological feedback are crucial for processes such as gene expression, metabolic regulation, and adaptation to environmental changes. Despite the prevalence of feedback in natural biological systems, its properties are not fully understood and moreover, its similarities and differences with engineered feedback remain understudied.^9,10^ In this work, we study the robustness of biological feedback to variations in its biochemical implementation and we quantify its system design properties.

In mechanical and electrical systems, feedback can be used to improve a system’s robustness against disturbances.^11^ For example, negative feedback in electronic circuits can improve robustness for a specific input range though it can also reduce overall gain and introduce fragility elsewhere. Indeed, it is well-known in engineering that feedback cannot provide complete robustness against all possible disturbances and that the use of feedback can introduce fundamental limitations on the system’s performance.^12,13^ Thus, the use of feedback in engineering can create performance trade-offs in terms of speed, stability, and accuracy that must be carefully balanced during system design.

In contrast, it remains mostly unknown whether feedback introduces similar trade-offs in biological systems.^14^ Previous research on this topic has experimentally validated a trade-off between efficiency and robustness in the glycolysis pathway of *Saccharomyces cerevisiae*.^15^ Other trade-offs were uncovered through modeling and mathematical analysis.^16−20^ However, we are still missing a general framework to study biological feedback and the trade-offs it may introduce.

A second key difference between feedback in engineered versus biological systems is that inside cells, feedback is implemented with biochemical reaction rates that can vary across many orders of magnitude and that can change due to mutations, and internal and external stimuli.^21^ Even in synthetically engineered biological circuits, the biochemistry of a feedback loop can be altered by its genetic, cellular, and extracellular contexts.^22,23^ Largely, it remains unclear how robust biological feedback is to variations in the biochemical reaction rates that implement it.^10,24^

In this paper, we quantify the robustness of five well-known models of biological motifs to variations in their biochemical parameters using sensitivity analysis. Our motivation for selecting these five motifs is that they represent simple synthetic circuits with feedback regulation that have been both modeled and experimentally constructed. First, we consider the mathematical models of positive and negative autoregulation,^1^ the toggle switch,^25^ the double-positive feedback motif,^26^ and the positive–negative feedback motif.^27^ We construct sensitivity functions that capture how each motif’s output changes with variations in its biochemical parameters. Subsequently, we pose and solve the multi-objective optimization problems of simultaneously minimizing pairs of sensitivity functions to determine the Pareto-optimal implementations of each motif.

We find that three of these biological examples are robust to variations in their biochemical parameters, while the negative autoregulation motif and the positive–negative feedback motif are constrained by robustness trade-offs. These results offer insight into how to optimally design robust, reliable synthetic circuits that can function robustly in changing environments.

The impact of our research is in providing a framework to study how robustness is allocated feedback in biological systems and what trade-offs are created by the use of biological feedback. Additionally, our research enhances our knowledge of systems biology and informs the system design of synthetic biological circuits.

## 2 Results and Discussion

### 2.1 Sensitivity Analysis

Intracellular feedback loops depend on physical parameters, such as the cooperativity and the binding affinity of biomolecules. Understanding how changes in these physical parameters change the concentrations of biochemical species in a system will give insight into the properties of feedback loops. Sensitivity analysis quantifies this relationship, revealing which parameters have the greatest impact on the behavior of a biological system.^2,28^

Throughout this paper, we will use sensitivity analysis to assess the robustness of biological systems with feedback to changes in their biochemical parameters such as binding and unbinding rates, cooperativities, and degradation rates. Here, we introduce the sensitivity function that we will use throughout the paper to determine how sensitive feedback loops are to variations in these parameters.

We assume that the biological systems we consider can be modeled using a system of nonlinear ordinary differential equations. The model is of the form:
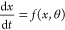
1where  is a vector of *n* biochemical species,  is a vector of *k* biochemical parameters, and *f* is a nonlinear, continuous function representing the dynamics of *x*. Assuming that a steady-state exists, we must have that
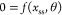
2where *x*_ss_ is the vector of biochemical species *x* evaluated at steady-state. The steady-state condition creates an implicit equation with variables *x*_ss_ and θ.

A simple measure of the sensitivity of biological systems to their biochemical parameters is given by the sensitivity function at steady-state. For a biochemical species *x*_ss_^*i*^ at steady-state, its sensitivity with respect to the parameter θ_*j*_ is given by
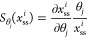
3Here, 1 ≤ *i* ≤ *n* and 1 ≤ *j* ≤ *k*. The sensitivity function can be thought of as a ratio between the fractional change in the biochemical species *x*_ss_^*i*^, given by , to the fractional change in its dependent parameter θ_*j*_. If *x*_ss_^*i*^ represents a biochemical species dependent on a parameter θ_*j*_, then  represents the ratio of the fractional change in the species to the fraction that we have perturbed the biochemical parameter from its original value by. Intuitively, the sensitivity function quantifies the percentage change in the species in response to a 1% change in the parameter.

To extend this idea further, the sensitivity function of a set of biochemical species at steady-state,  with respect to θ is simply
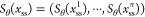
4

We briefly note that there are other methods to quantify the sensitivity of a system to variations in its parameters,^12,28^ but that we choose this local derivative-based sensitivity function for its simplicity and ease of application to nonlinear biological systems.

### 2.2 Pareto Optimality

Throughout this paper, we will use the multi-objective optimization (MOO) theoretical framework to simultaneously minimize multiple sensitivity functions. This will ensure robustness in our biological systems to variations in multiple of their biochemical parameters.

Multi-objective optimization (MOO) is a type of optimization problem in which more than one objective function is optimized at the same time. In MOO problems, the objectives can conflict with each other, meaning that improving one objective may lead to a deterioration in another. The goal of MOO problems is not to find a single “best” solution but rather a set of optimal solutions, known as Pareto-optimal, where no single objective can be improved without worsening at least one other objective. This set of optimal solutions is called the Pareto front.

We formulate the multi-objective optimization problem for our examples as follows:
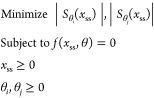
We assume that *x*_ss_ is the set of biochemical species at steady-state and that θ_*i*_ and θ_*j*_ are two of the biochemical parameters in the model in eqs 1 and 2. With this formulation, we aim to find the Pareto-optimal parameter set that simultaneously minimizes a pair of sensitivity functions.

We note that MOO problems have previously been solved for the system design of synthetic circuits^29^ and to understand other biological systems in refs (30−32).

### 2.3 Limits to Robustness in Biological Systems

#### 2.3.1 Positive Autoregulation Is Not Constrained by Trade-Offs

Positive autoregulation occurs when a gene or protein enhances its own production by stimulating its own expression.^1^ In this type of regulatory feedback loop, the product of the gene, such as a protein or RNA, increases the rate of its own synthesis, leading to the amplification of its expression. This mechanism helps sustain high levels of gene or protein expression, which is crucial for processes like cell differentiation, developmental pathways, and signal amplification in biological systems.^26,33,34^

The nondimensionalised model of positive autoregulation from ref (1) is given by the following ordinary differential equation in species *x*:
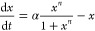
5

**Figure 1:**
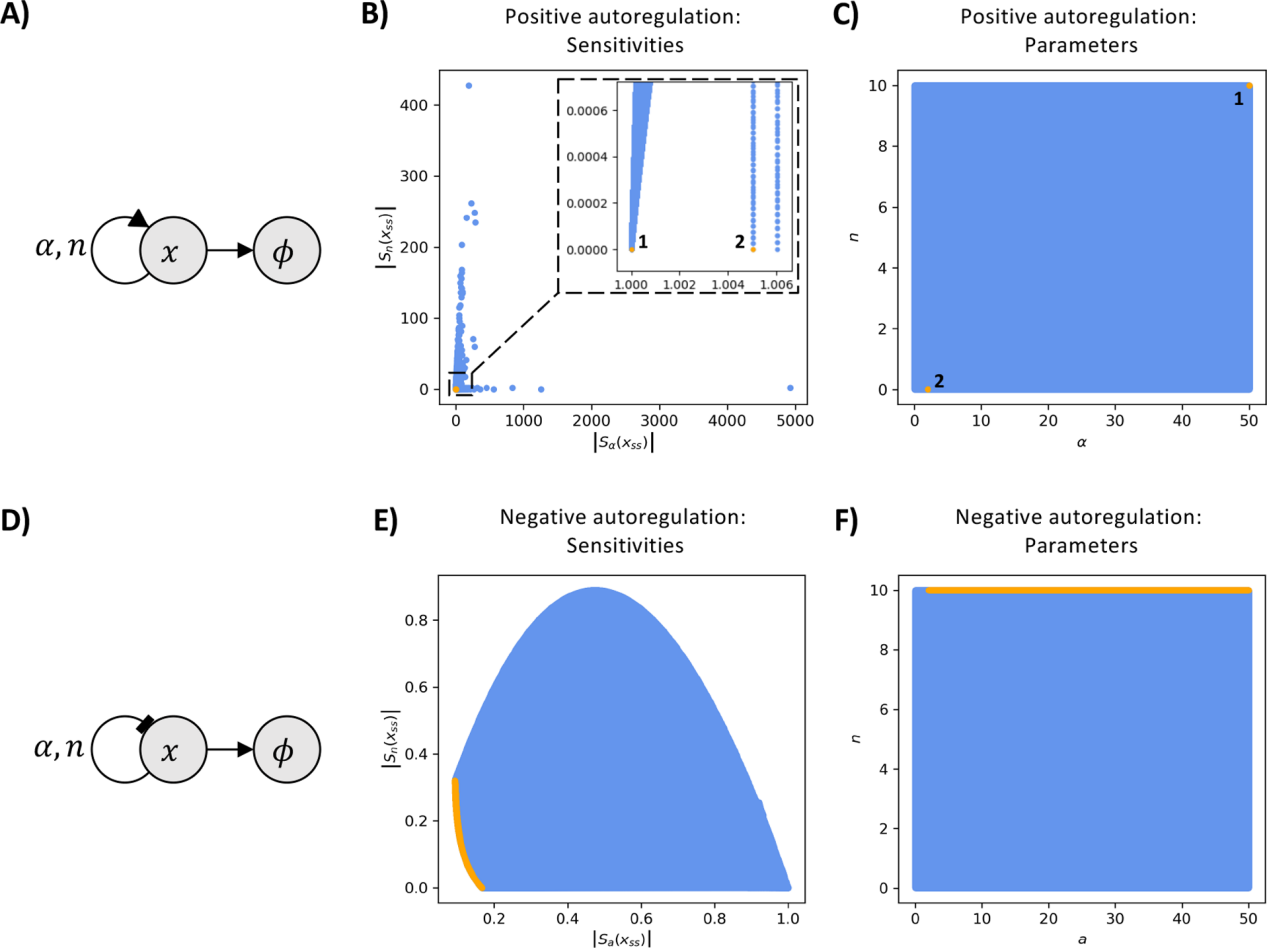
Pareto fronts of the sensitivity functions for the positive and negative autoregulation systems. Panels (A) and (D) show biological diagrams of the positive (A) and the negative (D) autoregulation of a biochemical species *x*. In panel (A), the pointed arrow represents positive feedback, where *x* enhances its own production, while in panel (D), the blunt arrow depicts negative feedback, where *x* represses its own production. We assume that species *x* also undergoes degradation and dilution within the cell, as indicated by the arrows pointed to the empty set symbol. The arrows indicate the parameter dependencies: *n* represents the binding cooperativity, while α represents the ratio of the production rate to the degradation rate per unit of dissociation constant concentration. We refer to α throughout the paper as the “feedback strength”. Panels (B) and (E) illustrate the Pareto fronts (orange) corresponding to the simultaneous minimization of the absolute values of the sensitivities with respect to the feedback strength, α, and to the cooperativity, *n* (blue). In panel (B), the Pareto front consists of two points, labeled 1 and 2. However, point 2 is identified as a numerical artifact of the sampling, meaning the true Pareto front contains only point 1. Solving the MOO problem shows that there is no trade-off between its two sensitivities. In panel (E), the Pareto front for the negative autoregulation system is represented by the orange curve. Solving the MOO problem shows that there is a trade-off between the sensitivities of the negative autoregulation circuit. Panels (C) and (F) illustrate the corresponding sampled parameters (blue) and the parameters responsible for the Pareto fronts (orange). In panel (C), two Pareto-optimal parameter pairs are identified for the MOO problem of positive autoregulation. However, since point 2 is a numerical artifact, only point 1 at maximal feedback strength and maximal cooperativity contributes to the Pareto front. In panel (F) the Pareto front is shown to be created by parameters with maximal cooperativity and feedback strength values of 2 or greater.

In this model, *n* represents the cooperativity constant of species *x*. Cooperativity is a molecular phenomenon where the binding of a molecule such as a ligand to a protein or enzyme influences the binding of additional molecules, either by enhancing or reducing more binding. Parameter α represents the ratio of the production rate to the degradation rate per unit of dissociation constant concentration. Throughout the paper, we refer to α as the “feedback strength”. Both parameters α and *n* are unitless quantities. We illustrate this biological circuit’s diagram in Figure 1A).

The steady-states of eq 5 occur when
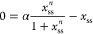
6

We note that *x*_ss_ = 0 is always a solution to eq 6. However, the sensitivity function  is ill-defined when *x*_ss_ = 0 due to division by zero. Hence the zero solution must be removed for our analysis. Thus, eq 6 reduces to
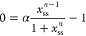
7

Equation 7 can have up to two solutions, depending on the choice of parameters. When one solution exists, it is always stable. When two solutions exist, one is stable and the other is unstable. We are only interested in the stable solution, as this is the one that can be observed and measured in practice.

In the Supporting Information, we provide the derivations of the relative sensitivities with respect to biochemical parameters α and *n*. The resulting sensitivity functions are:
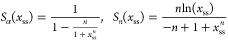
8

We note that the sensitivity function from eq 8. This indicates that changes in the feedback strength are always amplified by positive autoregulation. We solve the MOO problem of simultaneously minimizing the absolute values of these two sensitivities and illustrate the result in Figure 1B). We observe that there is no trade-off between the two sensitivities for the positive autoregulation circuit. This means that both sensitivities can be reduced at the same time without one negatively affecting the other. Intuitively, it makes sense that positive feedback ensures that small changes in parameters do not lead to large deviations from the desired steady-state. This analysis shows that positive autoregulation demonstrates remarkable flexibility because it can “tune” the two sensitivities independently, avoiding the typical conflict between them. With these properties, positive autoregulation can make clear and decisive responses that are particularly useful in biological processes that require an all-or-nothing decision.

#### 2.3.2 Negative Autoregulation Is Constrained by a Fundamental Trade-Off

Negative autoregulation is a regulatory mechanism in biological systems where a gene or protein inhibits its own expression.^1^ In this process, the product of a gene represses its own transcription, effectively reducing the level of its production. The role of negative autoregulation in biological systems is to quickly counteract changes in environmental conditions or cellular needs, helping to restore conditions.^1^

Previous research on the robustness of negative intracellular feedback has found that it is robust to variation in the cooperativity of the biochemical species, but sensitive to its production and degradation rates for specific values of these rates.^24^ We extend this result to reveal that a trade-off fundamentally constrains negative autoregulation. Specifically, we find that negative feedback cannot be robust to both variations in cooperativity and feedback strength at the same time.

We start by considering a system of one biochemical species undergoing negative autoregulation. We assume the species is produced via Hill kinetics with cooperativity constant *n* and that its feedback strength is α.^1^ This system is illustrated in Figure 1D) and modeled by the following nondimensionalized differential equation model:
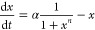
9

The steady state of this system occurs when
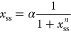
10

We note that eq 10 has a unique steady-state solution. We want to assess the sensitivity of this steady state to changes in each of its two dependent parameters: α and *n*. We will therefore inspect the following sensitivity functions:
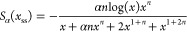
11
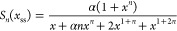
12

In Figure 1E), we note that there is a trade-off between the sensitivity to cooperativity and the sensitivity to feedback strength. This implies that the negative autoregulation system must have either some sensitivity to changes in feedback strength or to changes in cooperativity. Therefore, for a negative autoregulation system with cooperativity *n*, a higher value of the feedback strength will provide robustness to small changes in the feedback strength, and vice versa. To build a negative autoregulation circuit that is highly robust to changes in parameters, our modeling suggests that the feedback strength α should be greater than value two and the cooperativity *n* should be as high as possible. Clearly, finding the trade-off between the sensitivities provides valuable information about the system design of a robust negative autoregulation synthetic circuit.

#### 2.3.3 The Double-positive Feedback Circuit Is Not Constrained by Trade-Offs

A biological system with two positive feedback loops, such as those found in T-cell differentiation, can reinforce a precursor cell’s commitment to a specific lineage.^33^ These two feedback loops ensure a robust and irreversible decision, maintaining the differentiated state even after initial signals fade, and preventing partial differentiation. This design can be used in synthetic biology for stable state transitions or decision-making processes in cells.^34^

**Figure 2:**
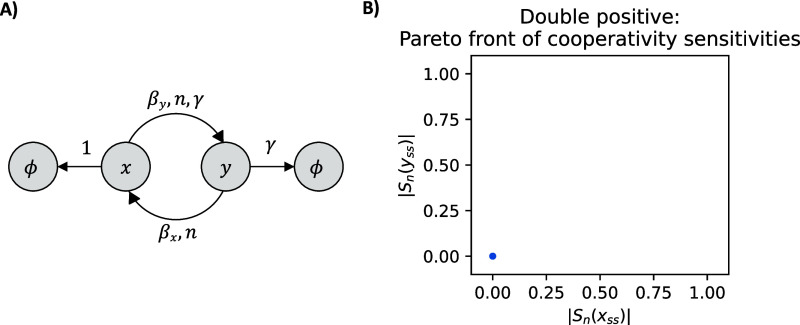
Diagram of the double-positive feedback system and the Pareto front of a representative pair of sensitivity functions. Panel (A) shows the diagram of the biological system with two positive feedbacks. The pointed arrows between species *x* and *y* represent positive feedback, where *x* enhances the production of *y* and vice versa. We assume that species *x* and *y* also undergo degradation and dilution within the cell, as indicated by the arrows pointed to the empty set symbol. The ratio of the rates of degradation and dilution is captured by the unitless parameter γ. After nondimensionalization, the species *x* is degraded at a rate of 1, β_*x*_ is the rate of production of species *x* and β_*y*_ γ is the rate of production of species *y*. Here, *n* represents the cooperativity of both species. Panel (B) illustrates the Pareto front corresponding to the simultaneous minimization of the absolute values of the sensitivities of species *x* and *y* with respect to the cooperativity *n*. The Pareto front for the positive-positive feedback system is the single point in blue. Solving the MOO problem shows that there is no trade-off between this pair of sensitivities. This result holds for all pairs of sensitivity functions, as plotted in Figure S3. The corresponding parameters are shown in Figure S4.

The double-positive feedback loop, illustrated in Figure 2A), consists of two mutually promoting species, *x* and *y*, that each undergo degradation. The nondimensionalized model of the double-feedback system can be described by the two ODEs in eq 13.
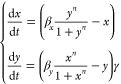
13

In this model, β_*x*_ and β_*y*_ are the ratios of the production rates to the degradation rates per unit of the dissociation constants for species *x* and *y*, respectively. The constant γ is the ratio of the degradation rate of species *x* divided by the degradation rate of *y*. The constant *n* represents the cooperativity of the two species and we assume it to be the same for simplicity. We note that β_*x*_, β_*y*_, γ, and *n* are unitless and we refer to β_*x*_ and β_*y*_ as the strengths of the two positive feedbacks.

The steady states for this system, , occur when:
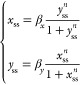
14

We note that the ratio of the degradation rates, γ, is not present in the steady-state equations. Plotting the nullclines in the parameter ranges that we consider biologically relevant reveals a single nonzero steady-state solution that is stable. It is the only solution we consider for this analysis.

The sensitivities functions of each steady-state species to parameters β_*x*_, β_*y*_ and *n* are given in Table 2. We note that due to symmetry, it must be that . In Figure 2B), we illustrate a representative result of the problem of simultaneously minimizing the absolute values of a pair of sensitivity functions. The Pareto front is comprised of a single point. This result holds across all pairs of sensitivity functions for combinations of both species (Supporting Information). Therefore, the double-positive feedback system is robust to changes in its biochemical parameters. This could be caused by the amplification created by the two positive feedbacks, mirroring the result of the positive autoregulation system.

#### 2.3.4 The Positive–Negative Feedback Circuit Is Constrained by Four Trade-Offs

The combination of positive and negative feedback is found in several biological processes such as the cell cycle. During the G2/M transition, positive feedback through cyclin-CDK complexes promotes rapid entry into mitosis, while negative feedback through checkpoint proteins such as p53 prevents progression if the DNA is damaged.^35^ The combination of positive and negative feedback is also believed to be responsible for robust oscillations found in natural systems. Synthetic circuits with these feedbacks were built in^7,36^

**Figure 3:**
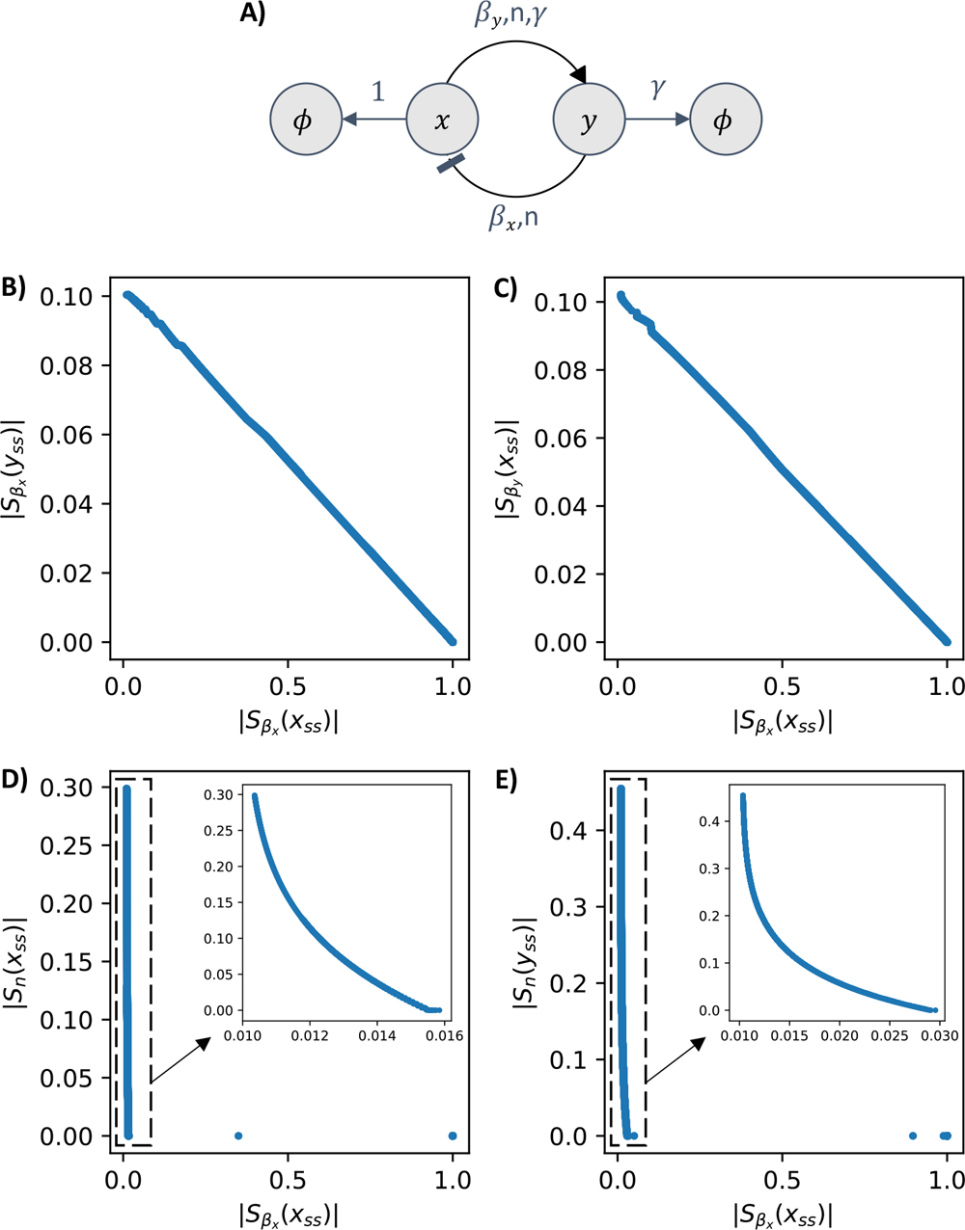
Diagram of the positive–negative feedback system and the Pareto fronts of four pairs of sensitivity functions. Panel (A) shows the diagram of a biological system with positive feedback (pointed arrow) from species *x* to species *y*, and negative feedback (blunt arrow) from species *y* to species *x*. Both species undergo degradation. After nondimensionalization, *x* is degraded at a rate of 1, *y* is degraded at a rate of γ, and production rates are β_*x*_ for *x* and β_*y*_ γ for *y*. The binding cooperativity is *n*. Panels (B–E) display the Pareto fronts obtained from the simultaneous minimizations of the absolute sensitivity of species *x* to its production rate β_*x*_ against four other sensitivities. The four Pareto fronts, shown as blue lines, highlight trade-offs between these four pairs of sensitivities. The arrows in panels (D) and (E) point to a zoomed-in view of the Pareto fronts. Solving the multi-objective optimization problems reveals four distinct trade-offs in the positive–negative feedback system. Additional plots of Pareto-optimal sensitivities and their parameters are found in Figures S5 and S6.

In the positive–negative feedback system, one species *x* promotes the expression of another species *y*, which in turn inhibits the production of *x*. We propose a simple mathematical model to capture these interactions. If we use similar biochemical assumptions to those in the derivation of the model for the double-positive feedback system, the dynamics of the positive–negative feedback system can be described by eq 15 and are illustrated in Figure 3A).
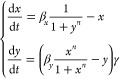
15

The steady states for this system, , occur when:
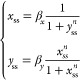
16

In this model, the nullclines intersect at a single steady-state solution, which is nonzero unless β_*x*_ = 0. Thus, within our parameter ranges of interest, a single stable nonzero steady-state is found. We think that the model would require additional complexity to produce the oscillations observed in the literature.^7,36^

For the single steady-state of the positive–negative system, we compute the sensitivity functions to variations in biochemical parameters and list them in Table 2. We note that due to symmetry, it must be that . In Figure 3, we can observe trade-offs between the sensitivity function  and all the other sensitivity functions: , and . There are no trade-offs for other pairs of sensitivities.

The simultaneous minimization of  and , as well as  and  yields Pareto fronts at very high cooperativity values for *n*. Thus, to design a robust positive–negative feedback circuit, increasing the binding cooperativity as much as possible is the best design choice.

#### 2.3.5 The Toggle Switch Is Not Constrained by Trade-Offs

**Figure 4:**
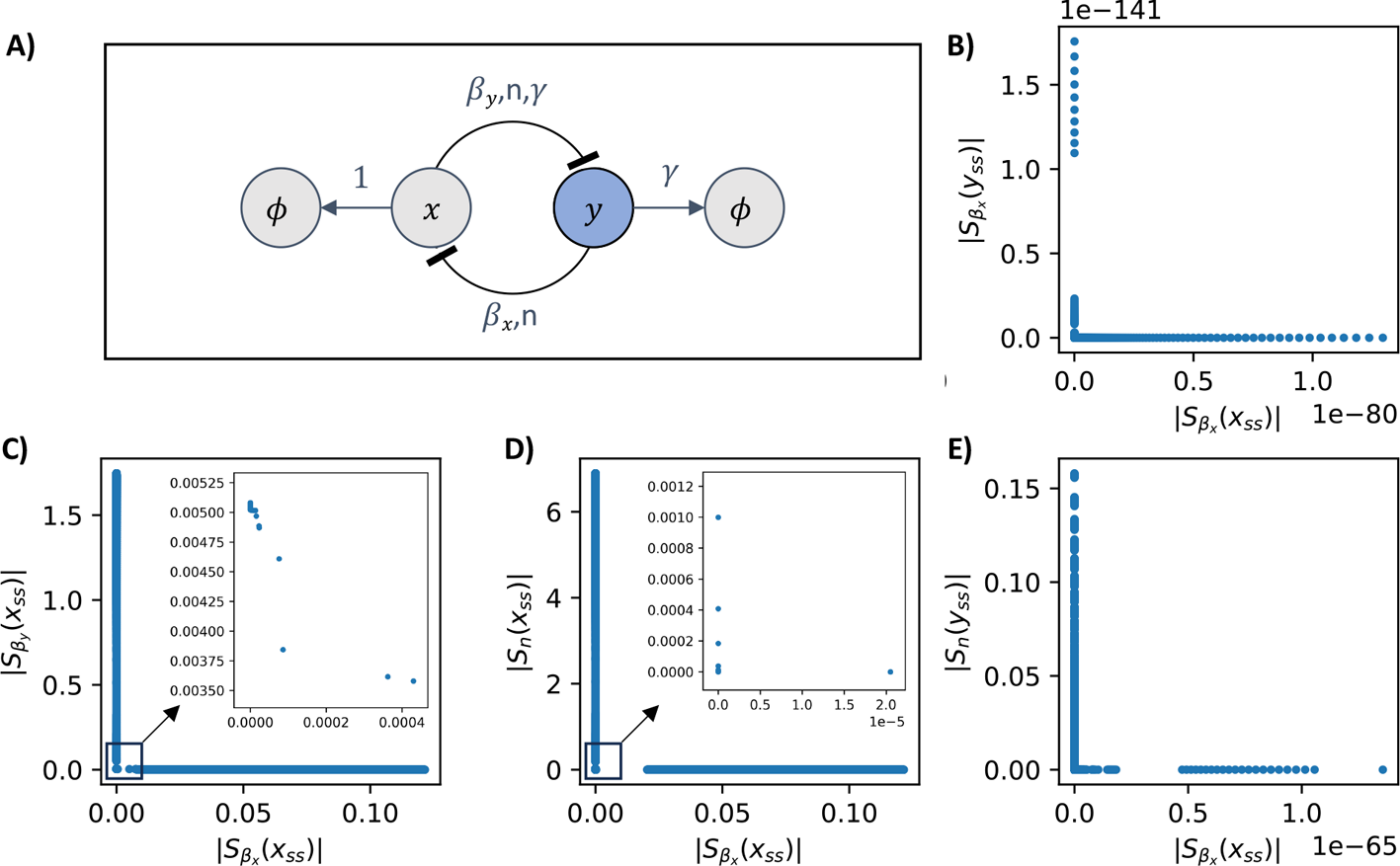
Diagram of the toggle switch and four of its Pareto fronts. Panel (A) shows the diagram of two biochemical species *x* and *y* that mutually inhibit each other. The inhibitions are illustrated by blunt arrows. Both species *x* and *y* are subject to degradation and dilution, as indicated by arrows pointing to the empty set symbol. The reaction rates are listed above each arrow. Panels (B–E) show the Pareto fronts obtained by simultaneous minimization of four sensitivity function pairs. In panels (C) and (D), we include the zoomed in version of the same figure. All four sensitivity function pairs are simultaneously minimized close to zero. Thus, no trade-offs are present in the toggle switch. Plots for all other sensitivities combinations and their corresponding parameters are included in Figures S7–S10.


The toggle switch is a classic synthetic circuit in which two species mutually inhibit one another while individually undergoing degradation and dilution.^25^ The toggle switch is a “winner takes all” system, where one species ends up depleted and the other plateaus to a higher steady-state concentration. The toggle switch, illustrated in Figure 4, can be described by the model in eq 17. This is a gene-interaction level model obtained from ref (37).

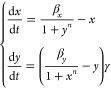
17The steady-states for this system, , occur when:
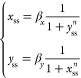
18

It is well-known that these two nullclines can intersect at up to three steady-state solutions. When three solutions are present, two are stable, and one is unstable. However, as the parameters change, the system undergoes a bifurcation where a stable and an unstable solution collide and disappear, leaving only a single stable solution. When only one stable steady-state exists, we compute its sensitivity function and later use it to supplement the two sensitivity functions that arise when two stable steady-states appear after the bifurcation.

The sensitivity functions associated with the stable steady-states of the toggle are given in Table 3. We note that due to symmetry, it must be that . In Figure 4, we illustrate representative results of the problem of simultaneously minimizing the absolute values of a pair of sensitivity functions. We direct the reader to the Supporting Information for the all the results of simultaneously minimizing pairs of sensitivity functions. All of these MOO problems are solved to find Pareto fronts close to value zero. Thus, the toggle circuit is unconstrained by trade-offs between its pairs of sensitivity functions.

### 2.4 Promoter Leakiness Reduces Trade-Offs across All Five Circuits

Previously, we considered ideal scenarios where gene regulation is unaffected by promoter leakiness. However, gene expression often occurs at a basal level due to promoter leakiness, where the promoter region allows for low-level transcription even when specific regulatory signals are absent.^1^ Interestingly, our research reveals that promoter leakiness can mitigate the previously discovered trade-offs. In this section, we consider the case of promoter leakiness for the negative autoregulation circuit. We assume that the promoter’s leakiness rate can range between 0 and 20% of the maximum expression of the promoter.^38,39^ This assumption should represent low and moderately leaky promoters. By denoting the promoter leakiness as the parameter *l*, the model of the negative autoregulation circuit is
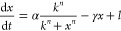
19Using the change of variables in Table 1, we arrive at the nondimensionalized model
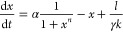
20The quantity  is a unitless value and so we can denote it as the nondimensionalised leakiness parameter *L*. The equation reduces to
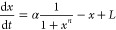
21As outlined in the Section 4, the parameters α, *k* and γ have ranges of interest of [0.02, 0.1] nM min^–1^, [0.01, 10] nM and [0.01, 0.24] min^–1^, respectively. We have assumed that the leakiness rate *l* can range between 0 and 0.2 α.^38^ Our range for the parameter *L* is therefore [0.002, 200].

**Table 1:**
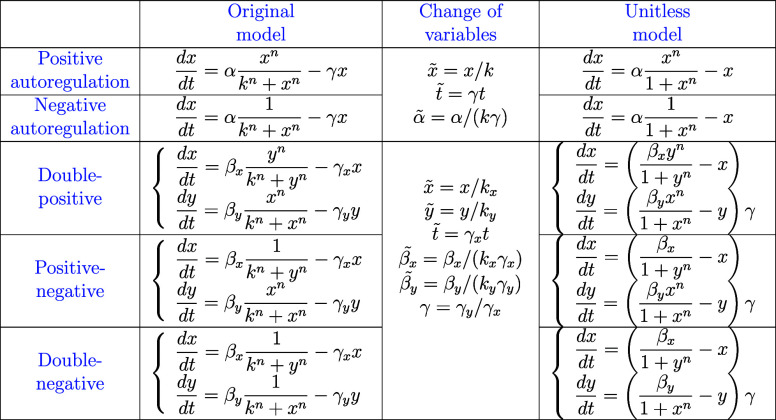
Mathematical Models Before and After Nondimensionalization for Each of the Biological Systems Studied in the Paper^a^

a Tildes denote the unitless analogue of their original physical quantity. For simplicity, we use the variables without tildes in the unitless models.

**Figure 5:**
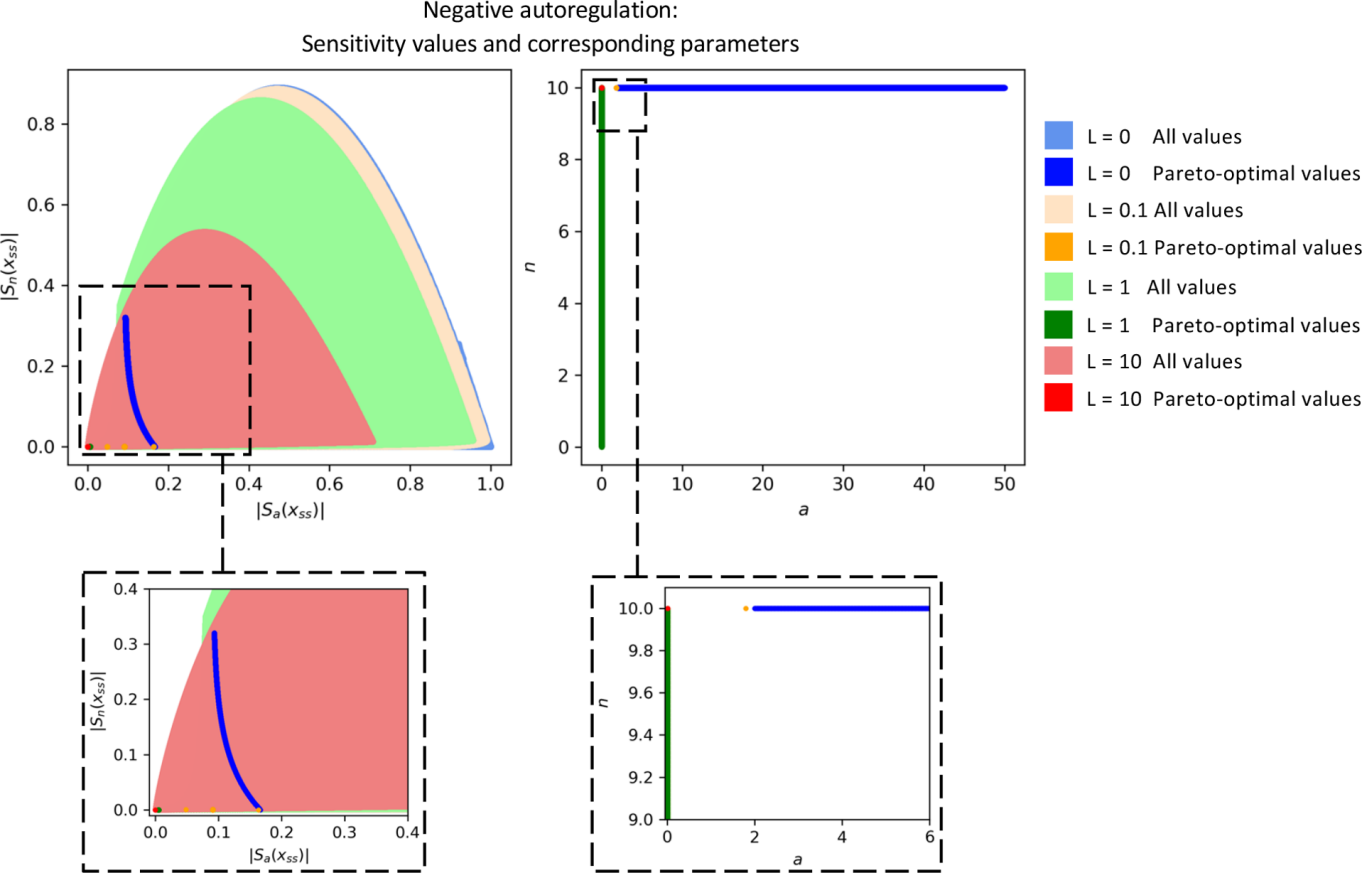
Increasing the promoter leakiness improves the trade-offs in the negative autoregulation circuit. We analyze four levels of leakiness: *L* = 0 (blue), *L* = 0.1 (orange), *L* = 1 (green), and *L* = 10 (red). In the left panel, sensitivity pairs  are plotted in lighter shades of their respective colors, forming contours that shrink as *L* increases. This shrinking parameter set illustrates how increased leakiness reduces sensitivity altogether. Correspondingly, the Pareto fronts for each leakiness level also collapse toward the origin, with the smallest sensitivity values observed at the largest leakiness level (*L* = 10). In the right panel, we show the parameter values corresponding to each Pareto front.

In Figure 1, we observed that negative autoregulation was constrained by trade-offs. Figure 5 highlights the effects of introducing promoter leakiness. The original result of *L* = 0 is shown in blue. At *L* = 0.1, with the same sampling of parameter pair (α, *n*), the area spanned by the sensitivity pairs  decreases slightly, and the Pareto front collapses drastically at the orange points. With each successive increase in the promoter leakiness *L*, the parameter region of the pair  shrinks further, and the corresponding Pareto fronts continue to move toward the origin, eventually eliminating trade-offs entirely.

This behavior can be explained as follows: as the leakiness constant *L* increases, the steady-state concentration of *x* rises. However, the rates d*x*/dα and d*x*/d*n* remain unaffected by the value of *L*. Consequently, the sensitivity functions  and  decrease. Thus, increasing promoter leakiness actually renders negative autoregulation more robust.

The same pattern holds true for the other four circuits examined. In each case, increasing promoter leakiness collapses any existing trade-offs between pairs of sensitivity functions into a single point. For further details on the effects of promoter leakiness, we refer the reader to the Supporting Information, where additional plots are provided.

Across all five synthetic circuits, we observed an absence of trade-offs between pairs of relative sensitivities when promoter leakiness is increased. Importantly, this result is not caused by the extensive optimization of the reaction rates corresponding to promoter strength, cooperativity, or production or degradation rates. By contrast, this result indicates that the combined regulatory mechanisms of feedback regulation and promoter leakiness inherently provide the decoupling of the sensitivities to variations in individual parameters. This result suggests that the combined regulatory mechanisms of feedback regulation and promoter leakiness provide flexibility to these circuits’ designs without the need for extensive optimization or fine-tuning.

We refer the reader to supplementary Figures S11–S14 for plots illustrating the effect of promoter leakiness on the positive autoregulation circuit, the double-positive feedback circuit, the positive–negative feedback circuit, and the double-negative feedback circuit.

### 2.5 Adding a Downstream Gene to the Positive Autoregulation Circuit Does Not Introduce New Trade-Offs

We aim to explore the question of whether circuits that are unconstrained by trade-offs remain so if they are connected to downstream processes. As a case study, we connect the positive autoregulation circuit to a downstream gene and evaluate the impact on the trade-offs of the autoregulated gene’s sensitivity functions. In this analysis, we assume that the downstream gene does not use up the species in the positive feedback circuit. The result is that the positive autoregulation circuit remains unconstrained by trade-offs even after the addition of the downstream gene.

**Figure 6:**
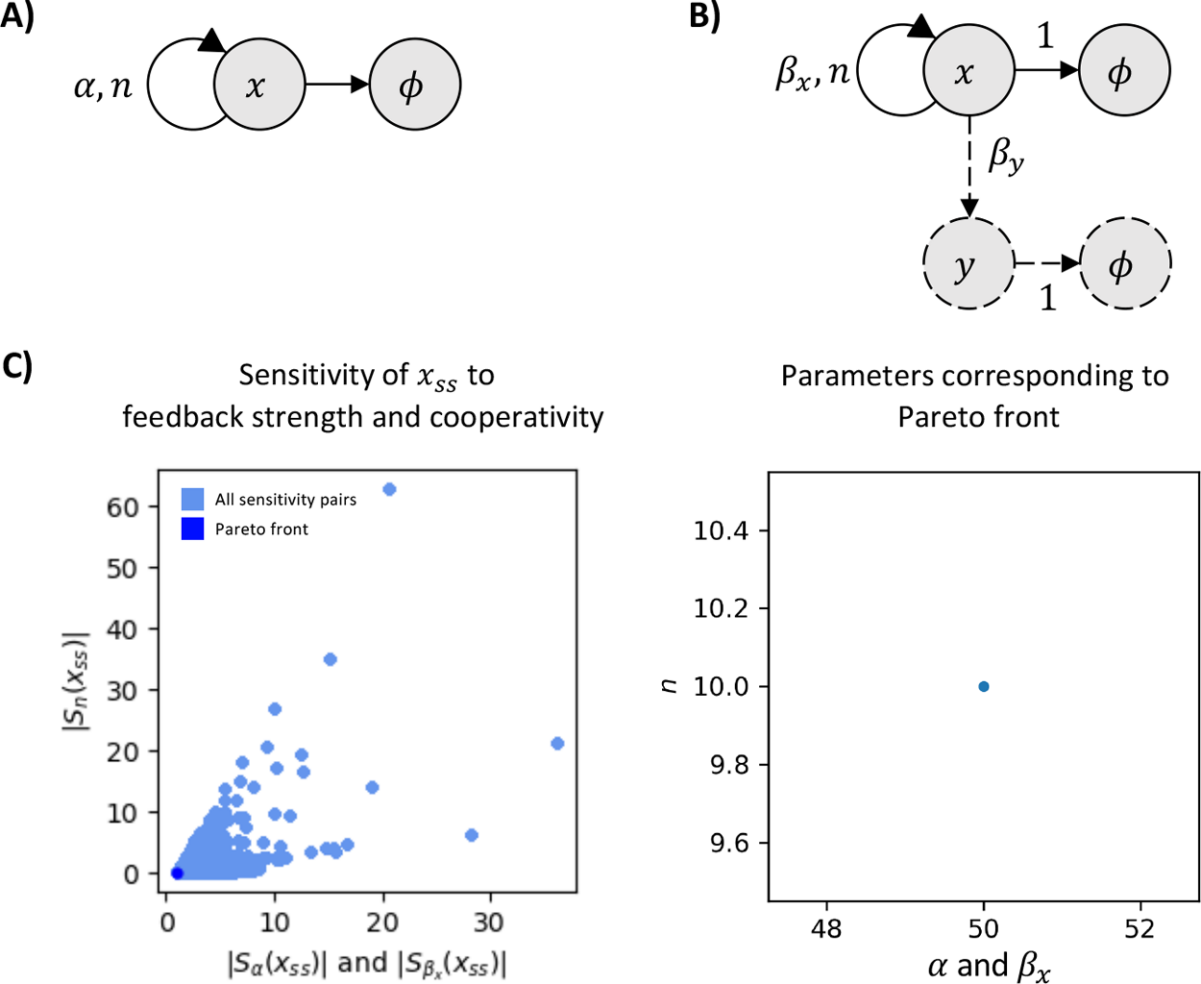
Adding a downstream gene to the positive feedback circuit does not introduce any new trade-offs. Panel (A) Diagram of a single positive autoregulating species. Panel (B) The same positive autoregulation circuit (drawn in solid lines) regulates a downstream gene (drawn in dashed lines). Panel (C) (Left) Plot of all the sensitivity values (light blue) and their Pareto front (dark blue) of the sensitivity of *x* at steady state to its feedback strength α for circuit in panel A and β_*x*_ for circuit in panel (B) and to its cooperativity constant (*n*). A single Pareto point at (1, 0) is observed, indicating the absence of a trade-off. (Right) The corresponding parameter for this Pareto-optimal point is at the maximal feedback strength and the maximal cooperativity: α, β_*x*_ = 50 and *n* = 10. The circuits in panels (A) and (B) share the same sensitivity functions.

To demonstrate this, we compare the trade-offs for a positively autoregulated gene *x* with production rate (α) and cooperativity (*n*) to a scenario where x produces an external gene *y* without incurring any cost to the *x* population. This comparison is depicted in Figure 6B), where the circuitry represented by solid lines corresponds to the autoregulation of *x*, and the circuitry represented by dashed lines illustrates the downstream components. The former represents the previously established positive autoregulation circuit, while the latter can be mathematically described as follows:
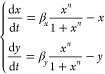
22

Here, the rate β_*x*_ represents the feedback strength of *x* (identical to α) and β_*y*_ represents the production rate of *y* driven by *x*. Both circuits have the same sensitivity values with respect to their respective feedback strengths and cooperativities. The sensitivity space is therefore identical and so are their Pareto fronts at  and *S*_*n*_ = 0. Since the inclusion of downstream gene *y* does not use up the species in the positive feedback circuit, the sensitivities and trade-offs of the combined circuit remain unaffected.

### 2.6 Positive Autoregulation Can Introduce New Trade-Offs in Downstream Gene Circuits, But It Can Also Enhance Their Robustness

**Figure 7:**
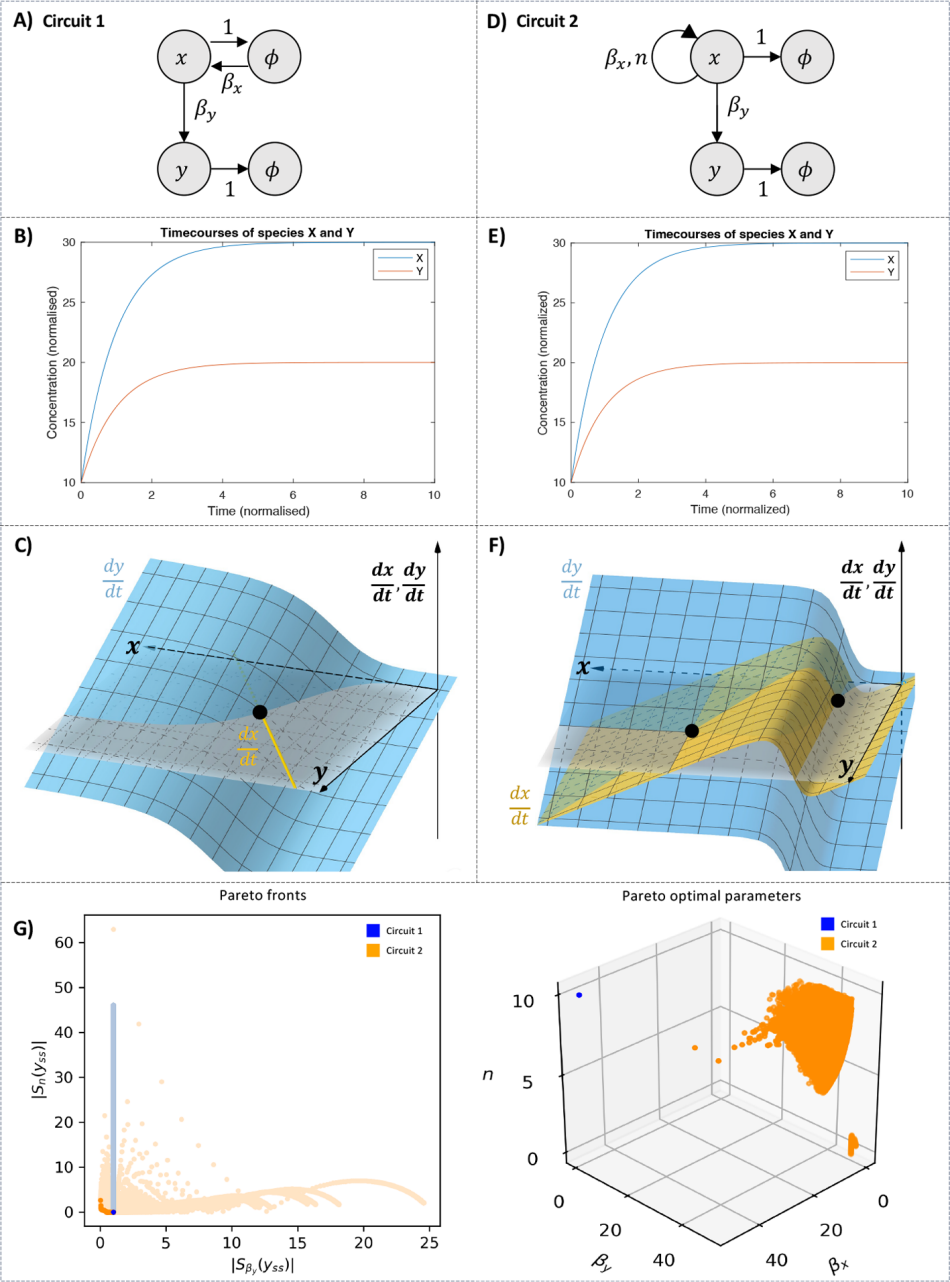
Introducing positive feedback upstream can create trade-offs for genes expressed downstream. Panel (A) depicts a constitutively expressed gene *x* regulating a gene *y*. The rate of species *x* expression is denoted by β_*x*_ and the rate of species *y* expression is denoted by β_*y*_. In Panel (D), the constitutive expression of *x* is replaced with a positive autoregulation of feedback strength β_*x*_ and cooperativity *n*. Panels (B) and (E) plot the timecourses of species *x* and *y* for identical conditions: *x*(0) = 10, *y*(0) = 10, β_*x*_ = 30, β_*y*_ = 20, and *n* = 10. Both circuits settle into stable steady-states. Panels (C) and (F) show that Circuit 1 only has one steady state (C) while Circuit 2 can have up to two steady-states (F). Panel (G) contains two plots. The left-hand plot shows the span of sensitivity function pairs  and  and their Pareto fronts. Circuit 1 is shown in blue and Circuit 2 in orange. Introducing positive autoregulation for species *x* creates a new trade-off (dark orange curve). The corresponding parameters for the Pareto front are shown in the right-hand plot.

As a case study on the impact of incorporating feedback into a circuit, we examine a two-species system in which gene *x* regulates gene *y*, and explore the effect of changing the expression of *x* from constitutive expression to positive autoregulation. We illustrate these circuits in Figure 7, in panels (A) and (D). The dynamics of the modified circuit are governed by the equations
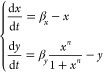
23while the dynamics of the latter are the same as in eq 22.

Without positive autoregulation, the sensitivities of the steady-state concentration of species *y* to its production rate (β_*y*_) and cooperativity constant (*n*) are unconstrained by a trade-off. The system exhibits a single optimal choice of parameters at which it is maximally robust. This is illustrated in blue in Figure 7G), where the light blue points in the left panel represent all sensitivities accessible to the system, and the dark blue point represents the Pareto front. When positive autoregulation is incorporated into dynamics of gene *x*, trade-offs emerge for the sensitivities of the steady-state species *y* with respect to β_*y*_ and *n*. This is shown in orange, where the light orange points in the left panel represent the new sensitivities spanned by sampling the same parameter space, and the dark orange curve represents the resulting Pareto front. This new Pareto front demonstrates that a trade-off now exists between the sensitivity to the production rate and the sensitivity to cooperativity. The system with positive autoregulation can access greater robustness to the production rate than in the previous circuit, but at the expense of increased sensitivity to cooperativity. Our findings suggest that positive autoregulation can enhance the robustness of downstream genes to variation in one parameter at the expense of increasing the sensitivity to another parameter, by introducing trade-offs that can access regions closer to the origin of the Pareto-optimality plot.

## 3 Conclusions

In this paper, we introduced a general computational framework to analyze how sensitive biological systems with feedback regulation are to variations in their biochemical parameters and we revealed the trade-offs that are present for these systems. Our computational framework used multi-objective optimization to simultaneously minimize the sensitivities of biological systems to variations in pairs of biochemical parameters. The results of the multi-objective optimization problems revealed whether trade-offs between the sensitivities to variations in pairs of parameters were present.

To illustrate this computational framework, we considered five examples of biological systems with feedback regulation and analyzed their sensitivities to variations in biochemical parameters. In single-species feedback, a trade-off between the sensitivity to feedback strength and to cooperativity was found for negative autoregulation but was absent for positive autoregulation. Indeed, we found that in negative autoregulation, decreasing the sensitivity to changes in cooperativity was only possible by increasing the sensitivity in feedback strength, and vice versa.

In dual-species feedback, we found several trade-offs between the strengths of feedback and the cooperativity of biomolecules. In double-positive feedback and in the toggle switch, no trade-offs arose. However, positive–negative feedback was constrained by trade-offs, requiring that the system design carefully balances robustness to feedback strength and cooperativity.

Although each of the five circuits had distinct functional roles, common features shared among them were observed. The net feedback sign of a circuit appeared to be related to the presence or absence of trade-offs. The net-positive feedback architectures (e.g., positive autoregulation, double-positive feedback, and double-negative feedback) were interrelated by their lack of trade-offs in sensitivity functions, while the net-negative architectures (e.g., negative autoregulation and positive–negative feedback) were interrelated by their trade-off constraints. This suggested that further research on more general net-positive feedback architectures might be fruitful for finding robust designs. Additionally, promoter leakiness was found to universally reduce trade-offs if they were present or to maintain the absence of trade-offs if none existed. The combination of promoter leakiness and feedback regulation demonstrated exceptional robustness and decoupled the sensitivities to variations in individual reaction rate parameters.

Overall, our work contributes to understanding trade-offs in the robustness of biological systems with feedback regulation. While it is well-known that engineered feedback is constrained by robustness trade-offs, as it cannot fully counteract all disturbances, similar insights for biological feedback systems remain underexplored. This paper helps bridge that gap by providing evidence of analogous trade-offs in biological contexts.

Future directions of this research include optimizing the sampling algorithm to efficiently solve multiple-objective optimization problems for parameter spaces in higher than three dimensions. We discuss the limitations of the grid-sampler and potential other algorithms in Section 4.4 of the Methods. Further research could replace the sensitivity functions evaluated at steady-state with the sensitivity functions for transient dynamics developed by Wu et al.^40^ and assess the robustness of biological systems such as oscillators.^7,41^ Lastly, further research could use this computational framework to research other examples of biological feedback systems and to develop theoretical arguments for why some examples of feedback introduce robustness trade-offs, while others do not.

Our current research contributes to advancing the understanding of biological feedback in natural systems and to improving system design for synthetic biological circuits. Ultimately, we aim for our work to facilitate future research aimed at unraveling the complexities of feedback in biological systems and at enhancing the reliability of engineered biological applications.

## 4 Methods

### 4.1 Nondimensionalization of the Mathematical Models

Nondimensionalising biochemical kinetics involves defining the relationships between physical quantities with units and their unitless analogues, and performing a change of variables of the original equations. Table 1 lists the model pairs for each feedback mechanism, and the associated changes of variables.

### 4.2 Parameter Ranges for the Models of the Biological Systems

In this work, we assume that the synthesis rates α, β ∈ [0.02, 0.1] nM min^–1^ according to values from,^42^ that dissociation constants *k* ∈ [0.01, 10] nM,^42,43^ and that the degradation and dilution rates γ ∈ [0.01, 0.24] min^–1^, as follows from the typical doubling times of bacterial and yeast cells.^44,45^

From Table 1, unitless feedback strengths (α∼, β∼) are defined as the ratio between the maximal production rate (α, β) and the product of dissociation constant (*k*) with degradation rate (γ). The range of values for the unitless feedback strengths is therefore α∼, β ∼ ∈ [1/120, 1000]. For practicality, we sample the range [0.01, 50].

Cooperative binding falls into four classes: binding that does not change the system dynamics (*n* = 0), binding that impedes further binding (0 < *n* < 1), binding that has no effect on subsequent binding (*n* = 1) and binding that promotes further binding (*n* > 1). At *n* = 0, the feedback disappears. Therefore, we consider *n* > 0. We set a lower bound of *n* = 0.01 for our numerical simulations. The upper bound was set to *n* = 10 to cover the single digit cooperativities that are common to biological systems.^46^

### 4.3 Numerical Methods

The equations for steady-states in Table 1 cannot typically be solved analytically to find their solutions *x*_*ss*_ and *y*_*ss*_. Instead, we employ scipy.optimize.fsolve from Python to solve these equations numerically. The parameter set we use was obtained by evenly grid-sampling the parameter ranges of α, β and *n*. Additionally, since we are only interested in the stable steady-states of our biological systems, we filtered out the unstable steady-states by imposing the condition that the real part of their eigenvalues evaluated at the solutions is less than zero. The resulting solutions and their respective parameter values were then used to evaluate the corresponding sensitivity functions via the analytically derived formulas. We refer the reader to the linked Mathematica files for the analytical derivations of the sensitivity functions.

In the MOO problems, the Pareto front was found using the Python package paretoset, based on the method proposed by ref (47).

### 4.4 Limitations of the Sampling Algorithm

The correct identification of the Pareto front is dependent on sufficiently sampling the two- or three-dimensional biochemical parameter space. The grid-search sampler we use is a computationally expensive method. For example, to find the Pareto fronts for the MOO problems in the positive–negative feedback system, we use 10^9^ samples for the three-dimensional grid.

The development of computationally efficient algorithms for solving multiobjective optimization problems remains an active area of research. More efficient methods such as Multi-Objective Simulated Annealing^48^ could be used in future work to reduce the number of samples required to find the Pareto fronts.

### 4.5 Sensitivity Functions

In this section, we include the analytical form of the sensitivity functions for all two-species examples in Tables 2 and 3.

**Table 2:** Sensitivity Functions of the Positive-Positive Feedback and the Positive–Negative Feedback Example Systems

Positive- Positive Feedback	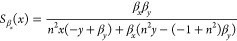
	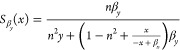
	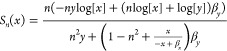
	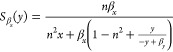
	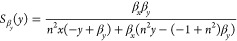
	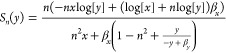
Positive-Negative Feedback	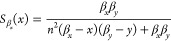
	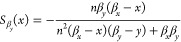
	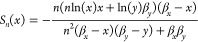
	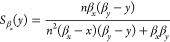
	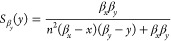
	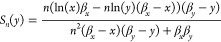

**Table 3:** Sensitivity Functions of the Toggle Switch

Negative- Negative Feedback	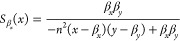
	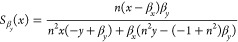
	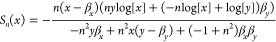
	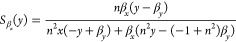
	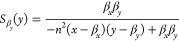
	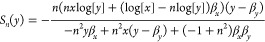

For brevity, we refer the reader to the code supplied in the GitHub repository for all sensitivity functions associated with leakiness from Section 2.4, and additional circuits covered in Sections 2.5 and 2.6.

## Data Availability

The Supporting Information includes additional figures as listed below. The manuscript figures, the Supporting Information figures, and the code used for figure generation are available on GitHub at the following link: https://github.com/nguyenhntran/ACSPaperSep2024.

## Supporting Information Available

Pareto fronts of double positive feedback (Figure S1); Pareto-optimal parameters of double positive feedback (Figure S2); Pareto fronts of positive–negative feedback (Figure S3); Pareto-optimal parameters of positive–negative feedback (Figure S4); Pareto fronts of double negative feedback at first equilibrium (Figure S5); Pareto-optimal parameters of double negative feedback at first equilibrium (Figure S6); Pareto fronts of double negative feedback at second equilibrium (Figure S7); Pareto-optimal parameters of double negative feedback at second equilibrium (Figure S8); effect of promoter leakiness on positive autoregulation (Figure S9); effect of promoter leakiness on double positive feedback (Figure S10); effect of promoter leakiness on positive–negative feedback (Figure S11); and effect of promoter leakiness on double-negative feedback (Figure S12) (PDF)
